# COVID-19-Associated Invasive Pulmonary Aspergillosis in the Intensive Care Unit: A Case Series in a Portuguese Hospital

**DOI:** 10.3390/jof7100881

**Published:** 2021-10-19

**Authors:** David Ranhel, Ana Ribeiro, Judite Batista, Maria Pessanha, Elisabete Cristovam, Ana Duarte, Ana Dias, Luís Coelho, Filipa Monteiro, Pedro Freire, Cristina Veríssimo, Raquel Sabino, Cristina Toscano

**Affiliations:** 1 Centro Hospitalar de Lisboa Ocidental, 1349-019 Lisbon, Portugal; acaribeiro@chlo.min-saude.pt (A.R.); mjlbatista@chlo.min-saude.pt (J.B.); mpessanha@chlo.min-saude.pt (M.P.); ecsantos@chlo.min-saude.pt (E.C.); aduarte@chlo.min-saude.pt (A.D.); apdias@chlo.min-saude.pt (A.D.); luismiguelcoelho16@gmail.com (L.C.); amonteiro@chlo.min-saude.pt (F.M.); pfreire@chlo.min-saude.pt (P.F.); ctoscano@chlo.min-saude.pt (C.T.); 2 Infectious Diseases Department, National Health Institute Dr. Ricardo Jorge, 1649-016 Lisbon, Portugal; Cristina.Verissimo@insa.min-saude.pt (C.V.); raquel.sabino@insa.min-saude.pt (R.S.); 3 Instituto de Saúde Ambiental, Faculdade de Medicina, Universidade de Lisboa, 1649-028 Lisbon, Portugal

**Keywords:** COVID-19, intensive care unit, *Aspergillus*, invasive pulmonary aspergillosis, COVID-19-associated invasive pulmonary aspergillosis

## Abstract

Invasive pulmonary aspergillosis (IPA) has become a recognizable complication in coronavirus disease 2019 (COVID-19) patients admitted to intensive care units (ICUs). Alveolar damage in the context of acute respiratory distress syndrome (ARDS) appears to be the culprit in facilitating fungal invasion in COVID-19 patients, leading to a COVID-19-associated pulmonary aspergillosis (CAPA) phenomenon. From November 2020 to 15 February 2021, 248 COVID-19 patients were admitted to our ICUs, of whom ten patients (4% incidence) were classified as either probable (six) or possible (four) CAPA cases. Seven patients had positive cultural results: *Aspergillus fumigatus* sensu stricto (five), *A. terreus* sensu stricto (one), and *A. welwitschiae* (one). Five patients had positive bronchoalveolar lavage (BAL) and galactomannan (GM), and two patients had both positive cultural and GM criteria. All but two patients received voriconazole. Mortality rate was 30%. Strict interpretation of classic IPA definition would have resulted in eight overlooked CAPA cases. Broader diagnostic criteria are essential in this context, even though differentiation between *Aspergillus* colonization and invasive disease might be more challenging. Herein, we aim to raise awareness of CAPA in view of its potential detrimental outcome, emphasizing the relevance of a low threshold for screening and early antifungal treatment in ARDS patients.

## 1. Introduction

Invasive pulmonary aspergillosis (IPA) is a well-defined issue in immunocompromised patients, especially in relation to hematologic malignancies and transplantation. However, the pathophysiology in the non-immunocompromised is different [1,2,3,4]. Acute respiratory distress syndrome (ARDS) due to severe viral infections, especially *influenza* disease, increases patients’ susceptibility to bacterial and fungal superinfections, including IPA [1].

This relationship between *influenza* and IPA has been sporadically conveyed in isolated cases over the past four decades. However, in the last decade or so, increasingly numerous reports and significantly robust cohort studies have shed light on *influenza*-associated pulmonary aspergillosis (IAPA) [5], which is currently a very well-known complication of severe *influenza* pneumonia with ARDS (19% incidence) [4]. There are several apparent factors involved in this pathogenesis, which are: direct damage to the epithelial–endothelial barrier of the pulmonary alveoli by respiratory viruses and inflammation, enabling *Aspergillus* to invade tissue; poor airway fungus clearance; immune dysfunction and dysregulation [1,5]—leading to an increased duration of hospitalization and mortality (with mortality rates of up to 51%) [4].

In December 2019, coronavirus disease (COVID-19) emerged from China and rapidly became a pandemic threat. Since then, there have been several reports of IPA in patients with COVID-19 admitted to ICUs, and similarly of *influenza*-associated pulmonary aspergillosis (IAPA). Furthermore, COVID-19-associated pulmonary aspergillosis (CAPA) has been recognized as a new entity [1,2,6,7,8,9,10,11,12], whilst ARDS, analogous to IAPA, appears to be the culprit in facilitating fungal invasion in CAPA [1,2].

Several of these cohort studies have reported CAPA in around 2.5% to almost as high as 40% of patients in the ICU [13], raising concerns about this superinfection as an additional contributing factor to mortality—especially considering the azole-resistant profile of *Aspergillus* mentioned in some reports [14,15].

## 2. Patients and Methods

We performed a retrospective observational study of all patients with COVID-19-associated ARDS admitted to the ICU at Centro Hospitalar de Lisboa Ocidental from 1 November 2020 to 15 February 2021 by reviewing microbiological and clinical data.

Detection of SARS-CoV-2 in respiratory specimens (nasopharyngeal swabs) was achieved by nucleic acid amplification test (NAAT) methods (Seegene STARlet^®®^, Bio-Rad CFX^®®^, Hologic Panther^®®^).

We evaluated patients based on the following: age, gender, preexisting conditions (immunocompromised and others), time between intubation and diagnosis of CAPA, time between corticosteroid therapy onset and diagnosis of CAPA, radiological findings, mycological data (respiratory specimen culture, molecular identification of the isolates, anti-fungal susceptibility testing), and serum/lower respiratory galactomannan index.

Microbiological evaluation of respiratory specimens involved microscopic examination of Gram- and Ziehl–Neelsen-stained slides and direct observation of growth media. Gram-stained slides were observed in all instances (allowing for sample quality assessment, semi quantitation of leukocytes, and morphological and Gram characterization of present microorganisms), and Ziehl–Neelsen-stained slides were observed depending on clinical information and/or underlying factors. Respiratory specimens other than bronchoalveolar lavage fluid samples (BAL) were plated on Columbia agar + 5% sheep blood, MacConkey agar and Chocolate agar PolyViteX with bacitracin, and observed daily during a 48 h incubation window (35–37 °C; 5–10% CO_2_). BAL samples were additionally plated on Sabouraud dextrose agar (30 °C; aerobiosis) and the incubation periods were longer, with a 72 h window for standard media, and 120 h window for Sabouraud dextrose agar (which could be prolonged to 8 weeks, if more exotic mycoses were suspected). Samples positive for filamentous fungi were re-plated to exclude cross contamination (specimens other than BAL would then also be plated on Sabouraud dextrose agar). Confirmed filamentous fungi were also subject to microscopic observation in lactophenol cotton blue stain.

Molecular identification of *Aspergillus* isolates was performed as described previously [16]. Antifungal susceptibility profiles were determined by broth microdilution reference method for susceptibility testing of molds. The M38-A2 protocol from the Clinical and Laboratory Standards Institute was applied for determining the Minimal Inhibitory Concentrations (MIC) for itraconazole, voriconazole and posaconazole, as previously performed [16].

To assess the severity of the disease, the Simplified Acute Physiology Score (SAPS II), Sepsis-Related Organ Failure Assessment (SOFA) and Acute Physiology and Chronic Health Evaluation (APACHE II) scores were used at ICU admission. The necessity of supportive therapy (vassopressive or inotropic agents and renal replacement) was also used as a severity criterion. To evaluate the severity of ARDS we used the Horowitz Index and the eventual need of mechanical ventilation, prone positioning and veno-venous extracorporeal membrane oxygenation (vvECMO).

Finally, regarding the diagnostic criteria of CAPA, patients were classified according to the revised definitions of invasive fungal disease of the European Organization for Research and Treatment of Cancer/Mycosis Study Group (EORTC/MSG) [17], the modified AspICU (modified) Algorithm [4] and the European Confederation for Medical Mycology and the International Society for Human and Animal Mycology Criteria (ECMM/ISHAM CAPA Criteria) [1].

## 3. Results

A total of 248 patients were admitted to our ICUs with COVID-19 in the aforementioned period (no cases of CAPA were found prior to November 2021), from whom resulted the presently described case series of 10 patients with mild to moderate ARDS and suspected diagnosis of CAPA (4% incidence) (Table 1).

Probable CAPA was diagnosed in six patients and possible CAPA was diagnosed in four patients, according to the most recent criteria for diagnosing IPA in COVID-19 patients in the ICU setting (ECMM/ISHAM CAPA Criteria) [1].

Demographic and clinical data were as follows: patients’ mean age of 65.8 years old, with a marginally increased prevalence in males (six males, four females). The most frequent comorbidities identified were chronic obstructive pulmonary disease (COPD), dyslipidemia, hypertension and obesity. The mean time between COVID-19 symptom onset and CAPA diagnosis was 18.9 days. All patients required mechanical ventilation and the mean time between intubation and CAPA diagnosis was 7.2 days.

Three of the patients took inhaled corticosteroids for COPD, and three other patients had previously received a solid organ transplant (SOT) (an EORTC host factor) and so were under a high-risk immunosuppressive therapy. Five patients received systemic corticosteroid treatment for COVID-19 (dexamethasone 6 mg q.d. or methylprednisolone 32 mg q.d.). Only one patient did not receive any dose of corticosteroids, for any length of time, and excluding the chronic users, the remaining patients were under immunosuppressive therapy for a mean of 17.67 days. None of the patients were treated with interleukin-6 (IL-6) inhibitors.

In total, 11 respiratory samples were studied—six BAL and five tracheal aspirates (TA) (one patient had both BAL and TA samples). Five cases were identified by positive culture. BAL galactomannan (GM) was tested in six of the ten patients, being positive in five of them (83% positivity), allowing for the identification of three cases by positive BAL GM alone, while the remaining two had both culture- and GM-positive criteria. There were no positive serum GM results. *Aspergillus* section *Fumigati* was the most frequently isolated species (*N* = 5), followed by *Aspergillus* section *Nigri* and *Aspergillus* section *Terrei* (*N* = 1 each). All *Aspergillus* isolated were proven to be susceptible to voriconazole treatment.

Almost all patients were given remdesivir (nine), and eight were given voriconazole. Therapeutic drug monitoring (TDM) of voriconazole was not made. Mortality rate was 30% (three patients died, and only two received antifungal therapy).

## 4. Discussion

IPA has been a well-defined complication, especially in immunocompromised patients. Although less frequent in non-neutropenic patients, these too are prone to facilitated fungal invasion in certain pathophysiological contexts. Severe *influenza* infection has already been described as intimately associated with IPA and, more recently, COVID-19-associated ARDS appears to provide the same alveolar damaging milieu that allows for invasive fungal disease, making CAPA an emerging serious secondary infection in COVID-19 patients.

Early diagnosis and treatment of CAPA are essential, considering the prognosis in untreated patients, with some studies mentioning excess mortality rates ranging from 16% to 25% [1] when compared to patients with no evidence of secondary aspergillosis infection. Nevertheless, diagnosis of IPA in this setting is as challenging as ever given the wide diversity of potential underlying conditions and pathophysiological nuances. Moreover, conflicting observations are to be expected regarding CAPA, stemming from differences in study design, testing methods and profiles, as well as case definition and criteria sets.

As indicated by early case series [1,2,3,4,6,7,8,9,10,12], and despite its usefulness in immunocompromised patients, IPA classification using EORTC/MSG criteria is associated with a high risk of missed diagnosis in non-neutropenic patients, since many of these might not have the required host factors and typical radiological features necessary for diagnosis. Moreover, an overlap of COVID-19 ARDS and invasive pulmonary aspergillosis radiological findings is highly likely, making it difficult to differentiate the two entities based on these findings. Alternatively, and according to the modified AspICU algorithm [4] and ECMM/ISHAM CAPA criteria [1], no such specific host factors are obligatory, and radiological findings, although still preferably reported by CT scan, can also be documented by chest X-ray. The diagnosis and distinction between putative and probable forms of CAPA results from the combination of radiologic abnormalities (pulmonary infiltrate or cavitating infiltrate not attributed to another cause) and mycological criteria (positive BAL culture, direct microscopic evidence of *Aspergillus* species in BAL or positive serum or BAL markers). The following are also considered as mycological criteria in the ECMM/ISHAM CAPA criteria: two or more positive *Aspergillus* PCR tests in plasma, serum or whole blood; a single positive *Aspergillus* PCR in BAL or a single positive *Aspergillus* PCR in plasma, serum or whole blood; and a single positive in BAL. Indeed, CAPA diagnosis would not have been possible in any but two of our patients, considering EORTC/MSG criteria, and adequate antifungal therapy would not have been initiated. In lieu of an absent gold standard—as lung biopsy is frequently not feasible in these critically ill patients—either the modified AspICU algorithm or ECMM/ISHAM CAPA criteria provided a much more adequate diagnostic framework, considering the non-neutropenic profile of these patients and the lack of those conventional host factors.

Admittedly, these are not definitive criteria, and the optimal approach for the diagnosis and subsequent management of CAPA will most likely involve a combination of different mycological criteria [18]. This will hopefully allow for some correlation with fungal burden, and concurrently minimize diagnosis resulting from single positive results which, as the criteria currently stand, are possible, and might otherwise just be the product of colonization or contamination during sampling and/or handling. Comparison studies between histopathological and mycological data are necessary in order to improve upon CAPA diagnostic tools and allow for proper evaluation of its real incidence and management.

Our incidence rate for CAPA (4%) was on the lower end of the spectrum observed in most recent cohort studies contemplating critically ill COVID-19 patients (ranging from 2.5% to 39.1%) [2,13]. There were probably other cases most likely undiagnosed mainly due to a lack of clinical awareness and diagnostic screening, especially owing to a decreased performance of invasive procedures and subsequent sample collection, which was necessary to avoid exposure from aerosolization and the associated high risk of transmissibility. Additionally, detection of *Aspergillus* in alternative specimens from the upper respiratory tract, such as sputum or tracheal aspirate, often does not permit distinction between colonization and invasive disease, harming the quality of mycological evidence and lowering diagnostic rates [1,2].

We found, besides corticosteroid administration, that dyslipidemia, hypertension, COPD and obesity were common characteristics among these patients, and the three COPD patients also had a history of smoking. These common comorbidities overlap with the ones mentioned in other studies [19]. While they might not be risk factors by themselves, they most certainly contribute to the overall poor clinical fitness of these patients, most likely adversely affecting their clinical outcomes. Mean APACHE II score, which is a marker of severity on ICU admission, was 25. Higher APACHE II scores, along with higher SOFA scores, also appear to be associated with IPA, and our results are similar to those found in other cohort studies [20].

Regarding positive isolates, the most common pathogen was *A. fumigatus* sensu stricto (71.4%), similar to other CAPA cohort studies [13] and to IAPA patients [4].

Furthermore, we found no positive serum GM in any of the cases, despite the presence of otherwise relevant positive criteria. Although positive serum GM is highly indicative of IPA, some studies suggest that serum GM might not be perfectly fitted as a criterion in non-neutropenic patients, considering its variable performance in this subgroup, and BAL GM might be more suitable for screening, due to a greater sensitivity [1,2,5,8]. However, whilst serum GM might be commonly negative in CAPA, including proven cases [1], this might not necessarily imply poor technical performance. Instead, it may represent a worse outcome at the end stage of the pathophysiological CAPA continuum—extending from colonization all the way to angioinvasion—with positivity possibly being indicative of higher mortality rates when directly comparing proven CAPA patients with and without positive serum biomarkers.

Regarding immunosuppressive therapy and excluding patients who had previously received an SOT and high-risk immunosuppressive therapy (conventional host factors), four patients received systemic steroids as adjuvant treatment for COVID-19 (three of which were already on inhaled steroids for COPD) during a mean of 7.4 days. Two of the three patients who died were on systemic corticosteroid therapy for COVID-19. The only patient who did not receive any immunosuppressive therapy is still alive (having received antifungal therapy) and one of the patients who died had not received antifungal therapy, despite having been treated with adjuvant immunosuppressants. While these are only highly anecdotal correlations, they beg the question whether severe COVID-19 itself is the main risk factor for a worse outcome, and whether it should be considered the highest treatment priority, or whether additional risk factors, such as potential superinfections such as CAPA, further and significantly increase this risk, and should instead be addressed with treatment precedence. It has been shown that chronic corticosteroid treatment is substantially more frequent in CAPA patients, and that the corresponding mortality rate is also higher [1]. Furthermore, it has long been established that high-dose and/or prolonged immunosuppressive treatments correlate greatly with increased risk and poor outcome of IPA [21]. As recent studies, such as the RECOVERY trial, demonstrated that corticosteroid therapy is beneficial in severe COVID-19 patients [22], it is to be expected that this leads to an increase in CAPA incidence, emphasizing the need for awareness and diagnostic and treatment standardization.

All but two of the patients were given voriconazole, the first-line and most effective treatment recommended for probable and proven CAPA [1]. There were no reports of drug–drug interactions, especially with remdesivir (also being a substrate for CYP3A4), which was given to nine of the patients (seven of which also received voriconazole). Even though possible CAPA does not constitute a diagnosis of certainty, it most likely benefits from antifungal therapy [1]. In certain geographical areas, testing for azole resistance is essential to adequate antifungal therapy due to increasing azole resistance rates, in which case voriconazole–echinocandin combination therapy or liposomal amphotericin B are the drugs of choice [14,15,23,24]. There was no known previous azole exposure in this cohort, and none of our *Aspergillus* isolates showed evidence of azole resistance.

Finally, our mortality rate was 30%, which is on the lower end of reported mortality rates from other cohort studies [4,13].

## 5. Conclusions

CAPA is a definite and worrisome complication in critically ill COVID-19 patients. This study demonstrates an incidence rate of 4% in the corresponding cohort. While it may seem low, it is not negligible and is most likely underestimated, which means it presents real detrimental potential for morbidity and mortality.

Through this report, we aim to raise awareness of CAPA and reinforce the value of early evaluation and prompt treatment of critically ill patients with COVID-19. Thus, we recommend testing for the presence of *Aspergillus*, ideally with bronchoscopy for collection of BAL, for fungal culture and GM measurement, and with GM measurement in consecutive serum samples, in all patients with COVID-19 admitted to the ICU who are worsening or not improving from their clinical condition.

## Figures and Tables

**Table 1:** Clinical characteristics of CAPA patients.

Characteristics	Patient 1	Patient 2	Patient 3	Patient 4	Patient 5	Patient 6	Patient 7	Patient 8	Patient 9	Patient 10
**Demographics**										
**Gender**	Male	Male	Male	Male	Female	Female	Female	Male	Male	Female
**Age (y)**	50	78	67	69	76	70	63	55	66	64
**Underlying Conditions**										
**Immunocompromised**	None	Inhaled steroids for COPD	Inhaled steroids for COPD + systemic corticosteroids (dexamethasone 6 mg q.d., 9 d)	Inhaled steroids for COPD + systemic corticosteroids (dexamethasone 6 mg q.d., 7 d)	Systemic corticosteroids (dexamethasone 6 mg q.d., 1 d)	High-risk immunosuppression	High-risk immunosuppression + systemic corticosteroids (dexamethasone 6 mg q.d., 5 d)	High-risk immunosuppression	Systemic corticosteroids (dexamethasone 6 mg q.d., 10 d + methylprednisolone 32 mg q.d., 5 d)	Prolonged use of corticosteroids (<0.3 mg/kg/d of prednisone)
**EORTC host factor**	None	None	None	None	None	SOT (2020/Dec): immunosuppressive therapy	SOT (2018/Dec): immunosuppressive therapy	SOT (2020/Dec): immunosuppressive therapy	None	None
**Other**	None	COPD, Alzheimer’s disease, osteoporosis, ex-smoker (for 17 y)	COPD, hypertension, hypothyroidism, ex-smoker (for >10 y)	COPD, obesity, hypertension, dyslipidemia, diabetes, 1st degree AV block, psoriasis, vitiligo, hepatitis B (resolved), ex-smoker (for >35 y)	Hypertension, dyslipidemia, hypothyroidism, basal-cell carcinoma (resolved), partial thyroidectomy and parathyroidectomy	Diabetes, paroxysmal atrial fibrillation, hiatal hernia, atypical gastric ulcer, degenerative spondylopathy, anemia	Left nephrectomy due to obstructive lithiasis, hypertension, hypothyroidism, dyslipidemia, hepatitis (undetermined etiology)	Hypertension, hypertensive nephrosclerosis, hypertensive cardiopathy, thyroidectomy and parathyroidectomy, cholecystectomy, hepatic steatosis	Morbid obesity (IMC 46), tibial fracture (resolved), lower extremity DVT	Sjögren syndrome, osteoporosis
**Time between symptom onset and CAPA diagnosis (d)**	10	17	23	16	12	16	11	7	42	35
**Time between ICU admission and CAPA diagnosis (d)**	7	2	11	8	1	16	5	3	39	4
**Time between intubation and CAPA diagnosis (d)**	7	2	11	8	1	16	5	3	17	2
**Time between corticotherapy onset and CAPA diagnosis (d)**	Not applicable	Chronic	11	Chronic + 7 (acute adjunctive)	1	37	5	17	35	Chronic
**Severity of Acute Illness at ICU Admission**										
**APACHE II score**	9	27	18	32	25	46	18	27	24	25
**SAPS II score**	16	63	33	75	58	88	40	33	43	66
**SOFA score**	11	11	10	7	11	Not determined	Not determined	Not determined	12	Not determined
**ARDS**										
**Horowitz index**	Mild (229 mmHg)	Moderate (145 mmHg)	Moderate (115 mmHg)	Moderate (175 mmHg)	Moderate (156 mmHg)	Moderate (144 mmHg)	Moderate (152 mmHg)	Moderate (148 mmHg)	Mild (271 mmHg)	Mild (276 mmHg)
**Mechanical ventilation**	Yes	Yes	Yes	Yes	Yes	Yes	Yes	Yes	Yes	Yes
**Prone positioning**	Yes	Yes	Yes	No	Yes	No	Yes	Yes	No	No
**vvECMO**	Yes	No	No	No	No	No	No	No	No	No
**Supportive Therapy**										
**Vassopressive or inotropic agents**	Yes (NA 3 cc/h)	Yes (NA 3 cc/h)	Yes (NA 5 cc/h)	Yes (NA 2 cc/h)	Yes (NA 3 cc/h)	Yes (NA 5 cc/h)	Yes (NA 3 cc/h)	Yes (NA 2 cc/h)	Yes (NA3 cc/h)	Yes (NA4 cc/h)
**Renal replacement**	CRRT	No	No	CRRT	No	No	No	No	CRRT	No
**Microbiology**										
**Fungal culture**	BAL: *Aspergillus* section *Fumigati*	TA: *Aspergillus* section *Fumigati*	TA: *Aspergillus* section *Fumigati*	TA: *Aspergillus* section *Fumigati*	TA: *Aspergillus* section *Terrei*	BAL: *Aspergillus* section *Fumigati*	BAL, TA: *Aspergillus* section *Nigri*	Negative	Negative	Negative
**Molecular identification**	*Aspergillus fumigatus sensu stricto*	*Aspergillus fumigatus sensu stricto*	*Aspergillus fumigatus sensu stricto*	*Aspergillus fumigatus sensu stricto*	*Aspergillus terreus sensu stricto*	*Aspergillus fumigatus sensu stricto*	BAL: *Aspergillus* *welwitschiae* (*A. niger* cryptic species)	Not applicable	Not applicable	Not applicable
**Susceptibility testing**	Susceptible to voriconazole, itraconazole and posaconazole	Susceptible to voriconazole, itraconazole and posaconazole	Susceptible to voriconazole, itraconazole and posaconazole	Susceptible to voriconazole, itraconazole and posaconazole	Susceptible to voriconazole, itraconazole and posaconazole	Susceptible to voriconazole, itraconazole and posaconazole	Susceptible to voriconazole, itraconazole and posaconazole	Not applicable	Not applicable	Not applicable
**BAL GM (≥1)**	Negative (<0.5)	Not determined	Not determined	Not determined	Not determined	Positive (1.0)	Positive (6.9)	Positive (1.3)	Positive (2.8)	Positive (1.0)
**Serum GM (≥0.5)**	Not determined	Negative	Not determined	Not determined	Negative	Negative	Not determined	Negative	Negative	Negative
**Definition of CAPA**										
**EORTC/MSG criteria**	Not classifiable (no host criterion)	Not classifiable (no host criterion)	Not classifiable (no host criterion)	Not classifiable (no host criterion)	Not classifiable (no host criterion)	Probable	Probable	Not classifiable (no clinical features—absent CT)	Not classifiable (no host criterion)	Not classifiable (no host criterion)
**AspICU (modified) Algorithm**	Putative	Colonization	Colonization	Colonization	Colonization	Putative	Putative	Putative	Putative	Putative
**ECMM/ISHAM CAPA Criteria**	**Probable**	**Possible**	**Possible**	**Possible**	**Possible**	**Probable**	**Probable**	**Probable**	**Probable**	**Probable**
**Imaging Studies**										
**Chest CT Scan**	Not available	Emphysema/pulmonary fibrosis lesions; ground-glass opacities with disseminated asymmetric and mainly peripheric consolidations	Not available	Not available	Not available	Multiple ground-glass opacities with apical-basal extension; moderate bilateral effusion	Disseminated extensive alveolar consolidations, and ground-glass opacities; minimal pleural effusion	Not available	Not available	Bilateral diffuse ground glass opacities; peripheral consolidation foci in the right lower lobe
**Chest Radiograph**	Bilateral pulmonary opacities	Not available	Bilateral diffuse interstitial infiltrates (peripheral)	Bilateral heterogenous infiltrates	Bilateral diffuse interstitial infiltrates	Not available	Bilateral diffuse hypotransparencies	Extensive bilateral hypotransparencies	Bilateral diffuse interstitial infiltrates (lower lobes)	Not available
**Therapy**										
**Antifungal (d)**	Voriconazole (8)	Voriconazole (8)	Voriconazole (7)	Voriconazole (17)	None	Voriconazole (8)	Voriconazole (14)	Voriconazole (3)	None	Voriconazole (10)
**Antiviral (d)**	Remdesivir (10)	Remdesivir (8)	Remdesivir (5)	None	Remdesivir (7)	Remdesivir (10)	Remdesivir (9)	Remdesivir (11)	Remdesivir (6)	Remdesivir (6)
**Outcome**	**Alive**	**Alive**	**Alive**	**Dead**	**Alive**	**Dead**	**Alive**	**Alive**	**Dead**	**Alive**

APACHE II—Acute Physiology And Chronic Health Evaluation II; ARDS—acute respiratory distress syndrome; BAL—bronchoalveolar lavage fluid; CAPA—COVID-19-associated pulmonary aspergillosis; COPD—chronic obstructive pulmonary disorder; COVID-19—coronavirus disease 2019; CRRT—continuous renal replacement therapy; CT—computed tomography; d—days; ECMM/ISHAM CAPA—European Confederation for Medical Mycology and the International Society for Human and Animal Mycology Criteria; EORTC/MSG—European Organization for Research and Treatment of Cancer/Mycosis Study Group; GM—galactomannan; ICU—intensive care unit; NA—noradrenaline; SAPS II—Simplified Acute Physiology Score II; SOFA—Sequential Organ Failure Assessment; SOT—solid organ transplant; TA—tracheal aspirate; vvECMO—veno-venous extracorporeal membrane oxygenation; y—years.

## Data Availability

Data presented in this study is not publicly available due to privacy restrictions. All data pertains to patient confidential clinical files and shall not be used to any other purpose.
